# Invasibility of Mediterranean-Climate Rivers by Non-Native Fish: The Importance of Environmental Drivers and Human Pressures

**DOI:** 10.1371/journal.pone.0109694

**Published:** 2014-11-05

**Authors:** Maria Ilhéu, Paula Matono, João Manuel Bernardo

**Affiliations:** 1 Departamento de Paisagem Ambiente e Ordenamento, Escola de Ciências e Tecnologia, Universidade de Évora, Évora, Portugal; 2 Instituto de Ciências Agrárias e Ambientais Mediterrânicas, Universidade de Évora, Évora, Portugal; University of Sydney, Australia

## Abstract

Invasive species are regarded as a biological pressure to natural aquatic communities. Understanding the factors promoting successful invasions is of great conceptual and practical importance. From a practical point of view, it should help to prevent future invasions and to mitigate the effects of recent invaders through early detection and prioritization of management measures. This study aims to identify the environmental determinants of fish invasions in Mediterranean-climate rivers and evaluate the relative importance of natural and human drivers. Fish communities were sampled in 182 undisturbed and 198 disturbed sites by human activities, belonging to 12 river types defined for continental Portugal within the implementation of the European Union's Water Framework Directive. Pumpkinseed sunfish, *Lepomis gibbosus* (L.), and mosquitofish, *Gambusia holbrooki* (Girard), were the most abundant non-native species (NNS) in the southern river types whereas the Iberian gudgeon, *Gobio lozanoi* Doadrio and Madeira, was the dominant NNS in the north/centre. Small northern mountain streams showed null or low frequency of occurrence and abundance of NNS, while southern lowland river types with medium and large drainage areas presented the highest values. The occurrence of NNS was significantly lower in undisturbed sites and the highest density of NNS was associated with high human pressure. Results from variance partitioning showed that natural environmental factors determine the distribution of the most abundant NNS while the increase in their abundance and success is explained mainly by human-induced disturbance factors. This study stresses the high vulnerability of the warm water lowland river types to non-native fish invasions, which is amplified by human-induced degradation.

## Introduction

The rate and extent of invasions in freshwater ecosystems are particularly alarming in the Mediterranean region, which is among the most heavily invaded ecosystems in the world [1]–[3]. The Iberian freshwater habitats are one of the most paradigmatic examples where there are constant reports of new invading fish species and colonization of new areas (see [4]–[10] as examples).

The success of biological invasions can be explained by several factors, with environmental drivers being among the most important ones [11]–[12]. One of the most frequently stated hypotheses in the biological invasion literature is that species should have a greater chance of success if they are introduced to an area with a climate that closely matches that of their original range [13]–[15]. Other environmental drivers, such as spatial heterogeneity and environmental variability, may also be important [16]–[18]. The success of an invading fish may be predicted with reference to environmental conditions at different scales, ranging from habitat to river types or ecoregions. Some of the features of the recipient ecosystem and the success of an introduced species may be determined by human-induced pressure. The “human activity” hypothesis argues that human activities facilitate the establishment of non-native species (NNS) by disturbing natural landscapes and by increasing propagule pressure [2], [8]. In Mediterranean-climate rivers, both landscape and human disturbance factors are expected to play a major role in the biological invasions, as these systems are largely governed by stochastic processes and have suffered a long history of human-induced pressure [19].

Although non-native species are not mentioned specifically in the European Union's Water Framework Directive (WFD) [20], in the context of the Directive's objectives NNS represent an important pressure (listed in the WFD annexes), since they can modify the structure of native biota and the ecological functioning of aquatic systems. NNS affect the ecological quality of natural environments in multiple ways and may represent a serious threat to native communities. Potential effects include genetic alterations within populations, spreading of pathogens and parasites, competition with and replacement of native species, and habitat deterioration or modification (see [21] for Iberian freshwater ichthyofauna). All the combined effects may result in changes in ecosystem function and global homogenization [1], [3], [22]–[23], with profound impact at ecological, evolutionary, genetic, and economic levels [24]–[25].

The apparently strong relationship between introduced fish species and degraded river conditions and their potential impact on native species and ecosystem suggest that NNS may be useful indicators of biological integrity and river health [26]–[28] and they are therefore incorporated in river monitoring schemes under the WFD and elsewhere. The incorporation of NNS into the ecological assessment will require knowledge about the density, distribution and potential risk of each species for each water body. According to the WFD, with the exception of particular cases, the ecological assessment involves the previous derivation of typologies for the aquatic systems. The ecological assessment is performed within groups of similar ecosystems and is expressed as a deviation from the reference conditions, i.e. conditions found in sites without human pressures. In fact, differences in climate, hydrology, geomorphology, geology, soil and vegetation make comparison of river communities difficult if not impossible. The use of river typology, by stratifying the spatial variability, makes the ecological assessment more practical, transparent and understandable by decision-takers, managers, technicians and public, and for these reasons it was the chosen approach on the WFD implementation. Moreover, approaching invasibility at a regional framework is useful for management purposes because it allows the identification of water bodies particularly vulnerable to NNS invasion and helps to regionalize monitoring schemes [29]–[31].

Understanding the factors promoting successful invasions is of great conceptual and practical importance. From a practical point of view, it should help to prevent future invasions and to mitigate the effects of recent invaders through early detection and prioritization of management measures. Thus, the identification of the environmental determinants of fish invasions is relevant in forecasting the overall impact of invasions on a global scale and prerequisite management authorities to adopt sound conservation policies.

The objectives of the present study were to determine: i) the patterns of non-native fish richness and abundance in Mediterranean-climate river types of Portugal; ii) the environmental drivers that favour the invasiveness of non-native fish within the morpho-climatic gradients; iii) the relationship between human-induced disturbance and non-native fish abundance and iv) the relative importance of environmental variables and human pressures for the occurrence and abundance of non-native fish species.

## Methods

### Study Area

**Figure 1:**
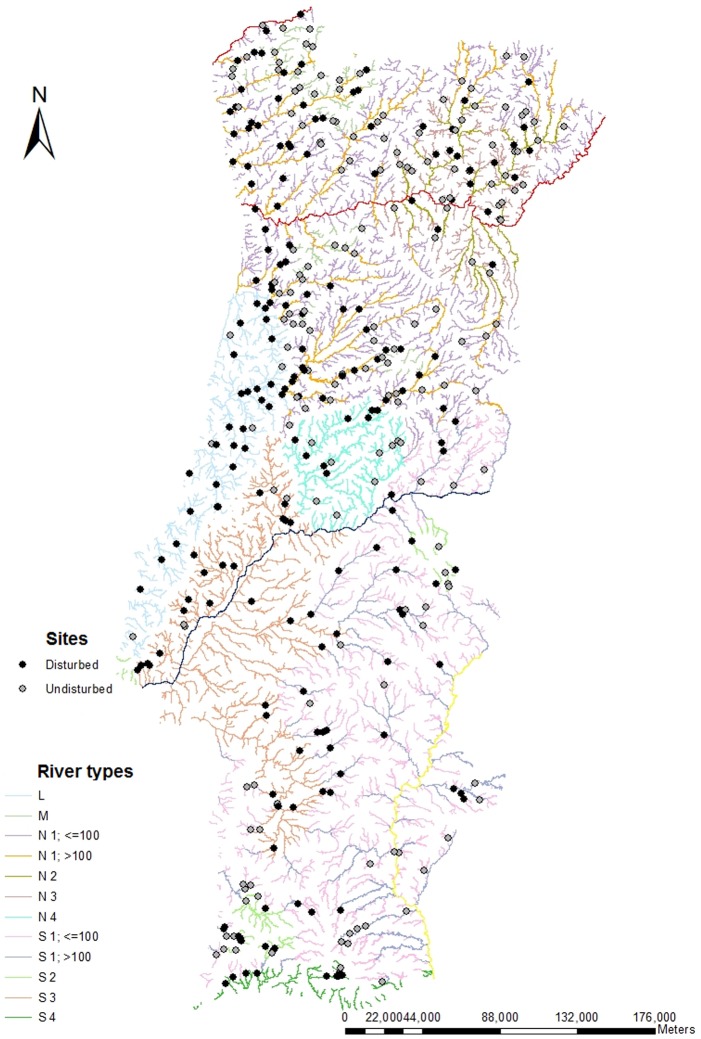
Map of the river types defined to continental Portugal showing undisturbed (grey dots) and disturbed (black dots) sampling sites.

The study considered 12 river types out of 15 defined for continental Portugal under the implementation of the WFD [32] (Fig. 1), as the 3 very large and highly modified river types were not considered.

The climate of Portugal presents high intra- and inter-annual precipitation and discharge variation, with severe and unpredictable floods between autumn and spring and persistent summer droughts [33], although the influence of factors such as topography and proximity to the Atlantic Ocean causes significant climatic contrasts. The general conditions of the atmospheric circulation cause a decrease in precipitation from north to south and from west (coast) to east (inland), enhanced by orographic asymmetry. Indeed, the mountain barrier in the north and center causes less rainfall in the interior regions. The temperature shows an opposite pattern, increasing from north to south. In a general way, the altitude causes a decrease in temperature and an increase in rainfall.

Portuguese river types reflect two main geographical and climatic gradients: north–south and west–east. The north–south gradient is associated with a decrease in altitude, precipitation, annual discharge, and increasing temperature. The west–east gradient is related to a continental effect, with a precipitation decrease and an increase in temperature extremes. Owing to these features, most rivers are permanent in the north and intermittent in the south. The most relevant climatic and morphological characteristics of the basins of the river-types are presented in Table 1 (see also [32]). The river types may be described as:

**Table 1:** Main morpho-climatic characteristics of river types in continental Portugal (mean and SD).

River-Types	Mean Annual Temperature (°C)	Mean Annual Precipitation (mm)	Altitude (m)	Drainage Area (km^2^)
**M**	11.0	1944	506	24.8
northern mountain streams	(1.5)	(379)	(300)	(17)
**N1<100 km^2^**	12.4	1190	413	33
north streams with small drainage area	(1.3)	(358)	(242)	(23)
**N1>100 km^2^**	12.6	1196	274	549
north streams with large drainage area	(1.2)	(374)	(205)	(65)
**N2**	13.1	596	300	960
streams from Alto Douro with large drainage area	(1.0)	(81)	(141)	(1115)
**N3**	13.0	671	432	32
streams from Alto Douro with small drainage area	(0.8)	(134)	(160)	(23)
**N4**	14.1	1065	280	151
transition streams between north and south	(0.7)	(168)	(122)	(361)
**L**	14.8	941	44	180
littoral streams from west/centre region	(0.3)	(118)	(44)	(671)
**S1<100 km^2^**	15.7	628	183	30
south streams with small drainage area	(0.9)	(86)	(75)	(21)
**S1>100 km^2^**	15.8	587	137	439
south streams with large drainage area	(0.9)	(84)	(68)	(579)
**S2**	15.4	743	175	60
southern mountain streams	(0.3)	(85)	(147)	(87)
**S3**	15.6	730	54	388
streams of sedimentary deposits in Tagus and Sado basins	(0.4)	(118)	(46)	(1081)
**S4**	16.9	632	54	67
southern carsick streams of Algarve	(0.5)	(60)	(57)	(89)

M - Mountain rivers of the North: trout rivers with small drainage area located in the North-West and North-Center regions with low mean temperature and high precipitation; N1<100 km^2^ - North rivers of small drainage area: low mean temperature, altitude mainly in the 200–600 m range; N1>100 km^2^ - North rivers of medium and large drainage area: characteristics similar to the previous type; N2 - Medium-large Alto Douro rivers: drainage area larger than 100 km^2^, high temperature (similar to the south types), lower rainfall; N3 - Small Alto Douro rivers: drainage area smaller than 100 km^2^ and characteristics similar to the previous type; N4 - North-South transition type: located at the center region with intermediate characteristics; L - Littoral type: coastal streams of small-medium dimensions located at the littoral center region, low altitude; S1<100 km^2^ - Small South rivers: a large group of low altitude temporary rivers, in a region of high temperature and low precipitation; S1>100 km^2^ - South rivers of medium-large dimension: larger drainage temporary rivers with similar characteristics to the previous type; S2 - South mountain rivers: small streams in the mountain regions of the south with lower temperature and higher precipitation than in the other south river types; S3 - Rivers of Tagus and Sado sedimentary basins: small and medium dimension, low altitude and high mineralization, smaller rivers with temporary regime; S4 - Small chalk rivers of Algarve: low altitude, low precipitation and high temperature.

Native freshwater fish fauna of Portuguese rivers presents relatively low species richness per site and most fish species are endemic cyprinids with high conservation value, particularly in the south. Many of those species are threatened with extinction [34]–[35].

### Sampling and data collection

Sampling was carried out between 2004 and 2006 during spring at 380 sites in the main Portuguese river basins (Fig. 1). The number of sampled sites in each river type was proportional to its basin area: M = 28 sites; N1<100 km^2^ = 60 sites; N1>100 km^2^ = 68 sites; N2 = 20 sites; N3 = 28 sites; N4 = 16 sites; L = 33 sites; S1<100 km^2^ = 26 sites; S1>100 km^2^ = 33 sites; S2 = 22 sites; S3 = 37 sites; S4 = 9 sites. In each river type, sampling sites included undisturbed or least disturbed sites and disturbed ones. For the site selection, a preliminary human-induced pressure screening using GIS and information on pollution loads was followed. The final selection was based on the human disturbance level regarding ten variables [36]–[37]: land use, urban area, riparian zone, connectivity of the river segment, sediment load, flow regime, morphological condition, presence of artificial lentic water bodies, toxic and acidification levels, and nutrient/organic load. The spatial scale of the assessment depended on the character of each pressure; some were made at local scale, others at the fluvial segment, and some others at the basin scale. Each variable was scored from 1 (minimum disturbance) to 5 (maximum disturbance) (Tables 2 and 3) and only sites with scores of 1 and/or 2 and only one variable scored with a 3 were considered as undisturbed or least disturbed (i.e., reference sites). The sum of these scores represents the total human pressure. Several water quality variables complemented the evaluation of human pressure in each site, following Standard Methods for the Examination of Water and Wastewater [38]: Biochemical Oxygen Demand (BOD_5_, mg L^−1^), Chemical Oxygen Demand (COD, mg L^−1^), Total Suspended Solids (TSS, mg L^−1^), Soluble Reactive Phosphorous (SR-P, mg L^−1^), nitrite (NO_2_^-^, mg L^−1^), nitrate (NO_3_^-^, mg L^−1^), ammonium (NH_4_^+^, mg L^−1^), and total inorganic dissolved nitrogen (N, mg L^−1^).

**Table 2:** Description, assessment scale and methods, and scoring criteria of the variables; land use, urban area, riparian vegetation, morphological condition and sediment load - used to evaluate the level of anthropogenic disturbance in sampled sites.

Variables	Description	Assessment scale	Score	Criteria	Methods
**Land use**	Impact of farming/forestry practices	River segment	5	>40% Agricultural use (intensive agriculture), very severe impact (rice field)	Local expert assessment complemented with Corine Land Cover (2000, 2006)*
			4	>40% Strong impact (area with strong forestry, including clearcuts)	
			3	<40% Moderate impact (subsistence gardens, pastures)	
			2	<40% Small impact (cork and holm oaks, high-growth forest)	
			1	<10% No significant impacts (natural forest and bush)	
	Land cover and bankface characterization	Local	5	Irrigated crops and/or high stocking	Local expert assessment complemented with Corine Land Cover (2000, 2006)*
			4	Horticultural crops, semi-intensive grazing	
			3	Extensive cultures (e.g. pastures, cereal crops, pine, eucalyptus), extensive grazing	
			2	Cork and holm oaks	
			1	Natural	
**Urban area**	Impact of urban areas	River segment	5	Very severe (location near a city with basic sanitation needs)	Local expert assessment complemented with Corine Land Cover (2000, 2006) *
			4	Town	
			3	Village	
			2	Hamlet	
			1	Negligible (isolated dwellings)	
**Riparian vegetation**	Deviation from the natural state of the riparian zone	River segment	5	Lack of riparian shrubs and trees (only the presence of annual plants)	Local expert assessment
			4	Fragmented vegetation with bushes and/or the presence of reed	
			3	Second replacement step (dominance of dense brushwood)	
			2	First replacement step (presence of shrub or tree strata with some level of preservation).	
			1	Potential vegetation (presence of shrub and tree strata according to the geo-series)	
**Morphological condition**	Deviation from the natural state of the stream bed and banks	Local	5	Transverse and longitudinal profile of the channel completely changed, with very few habitats	Local expert assessment
			4	Channelized sector, missing most of the natural habitats	
			3	Channelized sector, missing some types of natural habitats, but maintaining much of the shape of the natural channel	
			2	Poorly changed sector, close to the natural mosaic of habitats.	
			1	Morphological changes absent or negligible	
**Sediment load**	Deviation from the natural sediment load (both carried in the water column and deposited on the riverbed)	River segment and local	5	>75% of coarse particles of the stream bed are covered with fine sediments (sand, silt, clay)	Local expert assessment
			4	50–75% of coarse particles of the stream bed are covered with fine sediments (sand, silt, clay)	
			3	25–50% of coarse particles of the stream bed are covered with fine sediments (sand, silt, clay)	
			2	5–25% of coarse particles of the bed are covered with fine sediments (sand, silt, clay)	
			1	<5% of coarse particles of the stream bed are covered with fine sediments (sand, silt, clay)	

*information available from http://sniamb.apambiente.pt/clc/frm/.

**Table 3:** Description, assessment scale and methods, and scoring criteria of the variables: hydrological regime, toxic and acidification levels, organic and nutrient loads, artificial lentic waters - used to evaluate the level of anthropogenic disturbance in sampled sites.

Variables	Description	Assessment scale	Score	Criteria	Methods
**Hydrological regime**	Deviation from the natural hydrological regime (flow pattern and/or quantity). Includes all sources of hydrologic alteration, such as significant water abstraction	Local	5	<50% and strong deviation from the natural variability of the flow regime	Local expert assessment complemented with SNIRH
			4	<50% and moderate deviation from the natural variability of the flow regime	
			3	>50% and duration of flood periods close to the natural	
			2	>75% and duration of flood periods close to the natural	
			1	>90% and normal duration of natural flood periods	
		Local	5	<10% of mean annual discharge	Local expert assessment complemented with SNIRH
			4	<15% of mean annual discharge	
			3	>15% of mean annual discharge	
			2	>30% of mean annual discharge	
			1	>90% of mean annual discharge	
**Toxic and acidification levels**	Deviation from the natural state of toxicity conditions, including acidification and oxygen levels	Local	5	Constant for long periods (months) or frequent occurrence of strong deviations from natural conditions (e.g. pH <5.0, DO <30%)	Local expert assessment complemented with SNIRH
			4	Constant for long periods (months) or frequent occurrence of strong deviations from natural conditions (e.g. pH <5.5, DO <30–50%)	
			3	Occasional deviations (single measurements or episodic) in relation to natural conditions (e.g. pH <5.5, DO <30–50%)	
			2	Occasional deviations (single measurements or episodic) in relation to natural conditions (e.g. pH <6.0)	
			1	Conditions within the normal range of variation	
**Organic and nutrient loads**	Deviation from the normal values of BOD, COD, ammonium, nitrate and phosphate concentrations	Local	5	>20% of values in classes D or E	SNIRH (classification of water quality for multiple uses, according to the guidelines from the National Water Institute*), complemented with local expert assessment
			4	>10% of values in classes D or E	
			3	>10% of values in class C	
			2	No obvious or too small signs of eutrophication and organic loading	
			1	No signs of eutrophication and organic loading	
**Artificial lentic water bodies**	Impact related to the presence of artificial lentic water bodies upstream and/or downstream of the site (upstream change in thermal and flow regimes; downstream invasion by exotic species of lentic character)	Local	5	Local immediately downstream of a large reservoir or within the influence area of its backwater	SNIRH and available cartography
			4	Local immediately downstream of a mini-hydro or within the influence area of its backwater	
			3	Local downstream of a reservoir or within the influence area of the reservoir	
			2	Local downstream of a mini-hydro or within the influence area of its backwater	
			1	No influence of reservoirs	
**Connectivity**	Impact of artificial barriers to fish migration	River basin and segment	5	Permanent artificial barrier	SNIRH, available cartography, documental data and local expert assessment
			4	Occasional passage of some species	
			3	Passage of certain species or only in certain years	
			2	Passage of most species in most years	
			1	No barriers or existence of an effective pass-through device	

*information available from http://snirh.pt/snirh/_dadossintese/qualidadeanuario/boletim/tabela_classes.php.

Environmental characterization of sites was based on regional and local variables. Regional variables were obtained from digital cartography with free Internet access and included the drainage area of the basin upstream the site (km^2^), distance from source (km), altitude (m), slope (%), mean annual discharge (mm), mean annual air temperature (°C), and mean annual rainfall (mm). Rainfall, temperature, and flow variables were described from 30-year data series. Topographical variables were derived from a Digital Elevation Model (DEM), with a 90-m grid cell resolution (CGIAR-CSI 2005), using ArcMap 9.1. Local variables were assessed *in situ* at each sampling occasion: water temperature (°C), conductivity (µS cm^−1^), pH, dissolved oxygen (mg L^−1^), mean stream wetted width (m), maximum and mean water depth (m), mean current velocity (m s^−1^), dominant substrate class, adapted from the Wentworth scale [39]: 1 – mud and sand; 2 – gravel; 3 – pebble; 4 – cobble; 5 – boulders; 6 – boulders larger than 0.50 m, riparian vegetation (%), shadow (%), and proportion of different habitat types (pool, run, riffle).

Human disturbance level was evaluated *in situ* regarding the same ten variables described above for site selection. A total of 182 undisturbed and 198 disturbed sites were sampled covering the full human pressure gradient in each river type. The proportion of sites along the degradation gradient was sampled according to their availability within each river type.

Fish were collected by electrofishing following the WFD-compliant and CEN sampling protocol [40]–[41]. A backpack battery-powered electrofishing equipment was used, wading in shallow reaches (<1.2 m) or from a boat in deeper areas. The power and pulse frequency of the equipment were adjusted to ensure capture efficiency but also that fish were only stunned for a short period of time. Captured specimens were carefully handled, ensuring the minimum possible stress, and were quickly returned to the river. No specimens of endangered or protected species were injured or sacrificed.

The present study was part of a large programme coordinated by the National Water Institute (INAG, Instituto da Água, now part of APA, Agência Portuguesa do Ambiente). The National Institute for the Nature Conservation and Forestry (ICNF, Instituto de Conservação da Natureza e das Florestas (http://www.proforbiomed.eu/project/partners/institute-conservation-nature-and-forest-icnf) provided the necessary fishing permits. All sampling took place in national public rivers, which were under the jurisdiction of the National Water Institute and ICNF. As all the fish manipulation was restricted to the sampling procedures and animal care was taken into account, it was not necessary to obtain permission from the National *Animal Care* and Use *Committee* (Comissão de Ética, Bioética e Bem-estar Animal).

Captures were quantified as density (individuals/100 m^2^) and biomass (grams/100 m^2^). The degree of invasibility was measured using NNS richness and abundance.

A nonparametric ANCOVA performed using the rank transformation analysis of covariance evaluated the existence of significant differences in the density and number of NNS between river types [42], [43]. The total human pressure was included as a covariable, in order to exclude its effects from the analysis.

Mann-Whitney test was used to identify significant differences in the number and density of NNS between undisturbed and disturbed sites. The Z test of proportions [44] evaluated the existence of significant differences in the frequency of occurrence of NNS between undisturbed and disturbed sites.

The response pattern of the most abundant NNS to the human-induced pressure gradient was tested with a nonparametric ANCOVA (detailed above) [42], [43] of NNS density between five human pressure classes. The establishment of these five quality classes (High, Good, Moderate, Poor and Bad) for the stressor gradient (total human pressure) to which sites were assigned, followed the approach used in the REFCOND Guidance Document [37]. To account for the possible influence of the environmental variables, a Principal Components Analysis (outputs not shown) was performed in order to extract the most relevant environmental gradient (PCA1), which was then included as a covariable in this analysis. For each species, only data from river types with occurrences were considered.

Univariate Generalized Linear Models (GLM) were used for partitioning the variance [45] among three sets of explanatory variables - environmental, human-induced pressure and spatial trends on the occurrence and density of the most abundant NNS. For each species, only data from river types with NNS occurrences were considered. The set of environmental variables included altitude, mean annual discharge, drainage area of the basin, slope, mean annual temperature, mean water depth, mean stream width, mean current velocity, dominant substrate class, proportion of each habitat type, pH, dissolved oxygen and conductivity. The set of human pressure variables included BOD_5_, COD, TSS, N, SRP, and the 10 human pressure variables evaluated at each site (Table 2). The spatial structure of data was incorporated into the analyses to prevent misinterpretation of relations between and within the spatially arranged datasets [46]. Owing to spatial autocorrelation, values of particular variables in neighbouring sites are more or less similar than they would be in a random set of observations [47]. Autocorrelation is a frequently observed feature in spatially sampled biological data that may make the identification of plausible relationships between biota and the environment difficult [48]. The spatial structure of data was explored, including geographical coordinates of sites and their higher and cross product terms, in the modelling procedure (*x, y, xy, ×^2^, y^2^, x^2^y, xy^2^, ×^3^* and *y^3^*) [47]. The *x* and *y* coordinates were centred to zero mean before computing the matrix of spatial descriptors, to reduce collinearity between successive terms when fitting the polynomial. GLMs were performed using a forward selection procedure of the explanatory variables. The best models (minimal adequate) were estimated according to the lowest Akaike Information Criterion (AIC). Plots of residuals (not shown) were examined to complement AIC values and to confirm goodness-of-fit (see [49]). For the occurrence of species (presence–absence data), GLMs were performed using the binomial distribution and logit link function. In order to overcome the problems of distribution fitting resulting from the high number of absences in the density matrix, this continuous response variable was standardized to the maximum value for the species and converted into four classes: 0 (0% of individuals), 1 (0% - 10% of individuals), 2 (10% - 50%) and 3 (>50%). For this transformed density response variable (count data), GLMs were performed using the Poisson distribution and log link function. The existence of over-dispersion in data (variance higher than the mean; dispersion parameter>1) was checked during the analysis. If the value observed was higher than the threshold limit then quasi-binomial and quasi-Poisson distributions should be used, respectively. The total variation within the response variables was decomposed among the three sets of explanatory variables and the percentage of explained deviance calculated for eight different components [50]: (i) pure effect of environmental drivers, (ii) pure effect of anthropogenic disturbance, (iii) pure effect of spatial trends, (iv) combined variation due to the joint effect of environmental and anthropogenic components, (v) combined variation due to the joint effect of environmental and spatial components, (vi) combined variation due to the joint effect of anthropogenic and spatial components, (vii) combined variation due to the joint effect of the three components and (viii) variation not explained by the independent variables included in the analysis.

Multicollinearity among explanatory variables may result in the exclusion of ecologically meaningful variables from the models if another intercorrelated variable or variables happen to explain the variation better in statistical terms. Therefore, some of the most clearly intercorrelated variables (Spearman's rank correlation IrI>0.75; *P*<0.05) were initially excluded. The exclusion decision took into account the potential relevance of the variable in the occurrence and distribution of NNS. Furthermore variables were maintained in the models only if their addition did not cause any Variation Inflation Factor (VIF) to exceed 3.0, therefore ensuring that no covariation exists between the selected variables in the models.

Prior to the analyses, all data were either log (x+1) (linear measurements) or arcsin [sqrt(x)] (percentages) transformed to improve normality. Statistical analyses were performed using CANOCO 4.5, STATISTICA 6, PRIMER 6, SPSS 21 and BRODGAR 2.6 software applications.

## Results

### Patterns of richness, distribution and abundance of non-native species

A total of 41 fish species were captured, including 6 diadromous species and 10 NNS: pumpkinseed sunfish *Lepomis gibbosus* (L.), Iberian gudgeon *Gobio lozanoi* Doadrio and Madeira, mosquitofish *Gambusia holbrooki* (Girard), carp *Cyprinus carpio* L, goldfish *Carassius auratus* (L.), largemouth bass *Micropterus salmoides* (Lacépède), chameleon cichlid *Herichthys facetum* (Jenyns), black bullhead *Ameiurus melas* (Rafinesque), pike-perch *Sander lucioperca* (L.), and bleak *Alburnus alburnus* (L.) (Table 4).

**Table 4:** Frequency of occurrence (f.oc.), number of species (%) and density (mean and SD) (ind/100 m^2^) of non-native fish species (NNS) for total data (a), undisturbed (b) and disturbed (c) sites in each river-type (see Table 1) with NNS occurrence.

		NNS f.oc.	Total	N1<100 km^2^	N1>100 km^2^	N2	N3	N4	L	S1<100 km^2^	S1>100 km^2^	S2	S3
NNS f.oc.	a)	0.30		0.1	0.4	0.7	0.07	0.2	0.5	0.4	0.7	0.3	0.5
	b)	0.18		0	0.23	0.67	0.12	0.11	0	0.13	0.50	0.25	0.17
	c)	0.46		0.25	0.58	0.80	0	0.29	0.58	0.70	0.79	0.40	0.52
Nbr. NNS	a)		12.0	3.0	13.1	19.3	2.9	4.6	14.8	13.1	31.1	16.3	19.6
(%)			(21.0)	(9.2)	(19.3)	(14.2)	(11.2)	(10.0)	(19.6)	(23.2)	(30.1)	(31.1)	(25.9)
	b)		5.3	0	5.6	19.0	4.9	2.2	0	2.6	17.3	7.4	4.2
			(12.0)		(10.9)	(15.2)	(14.2)	(6.7)		(7.2)	(19.5)	(15.6)	(10.2)
	c)		18.2	6.4	19.0	20.0	0	7.7	18.8	29.8	41.2	27.0	22.6
			(25.2)	(12.7)	(22.4)	(12.6)		(13.1)	(20.3)	(30.1)	(32.8)	(41.7)	(27.0)
NNS Mean density	a)		3.6	2.3	4.8	1.2	0.07	0.3	9.3	3.6	6.4	1.1	6.3
(ind/100 m^2^)			(12.5)	(9.1)	(12.8)	(1.6)	(0.3)	(0.8)	(30.1)	(8.6)	(11.5)	(2.1)	(12.5)
	b)		0.5	0	0.4	1.2	0.1	0.3	0	1.6	1.4	0.7	0.2
			(1.7)		(1.2)	(1.8)	(0.5)	(1.0)		(4.4)	(1.8)	(1.6)	(0.4)
	c)		6.4	4.8	8.2	1.2	0	0.2	11.8	6.9	10.1	1.5	7.5
			(16.8)	(13.0)	(16.3)	(0.9)		(0.4)	(33.6)	(12.4)	(14.1)	(2.7)	(13.3)
*Lepomis gibbosus*		0.21	0.9	0.1	1.1	0.6	0.01	0.3	0.8	2.3	2.1	0.06	2.3
			(3.3)	(0.9)	(3.7)	(0.9)	(0.02)	(0.8)	(2.1)	(6.7)	(4.4)	(0.3)	(5.4)
*Cyprinus carpio*		0.03	0.1	0	0	0.01	0	0	0.09	0.01	0.9	0	0.08
			(1.6)			(0.01)			(0.4)	(0.06)	(5.5)		(0.4)
*Carassius auratus*		0.008	0.01	0	0.01	0.01	0	0	0	0	0	0	0.01
			(0.03)		(0.06)	(0.01)							(0.02)
*Gobio lozanoi*		0.14	1.8	2.2	3.4	0.6	0.07	0	7.5	0	0	0	1.6
			(10.9)	(9.1)	(10.6)	(1.2)	(0.3)		(29.9)				(6.7)
*Micropterus salmoides*		0.02	0.07	0	0.2	0	0	0	0.2	0	0.1	0.07	0.08
			(0.7)		(1.5)				(0.6)		(0.6)	(0.3)	(0.5)
*Herichtys facetum*		0.01	0.03	0	0	0	0	0	0	0.3	0.1	0	0
			(0.4)							(1.3)	(0.3)		
*Ameiurus melas*		0.003	0.1	0	0	0	0	0	0	0	0	0	1.2
			(2.2)										(7.1)
*Gambusia holbrooki*		0.11	0.5	0	0.03	0	0	0.01	0.7	1.1	2.7	0.9	1.1
			(3.1)		(0.2)			(0.06)	(2.0)	(2.5)	(9.0)	(2.1)	(3.2)
*Sander lucioperca*		0.003	0.01	0	0.04	0	0	0	0	0	0	0	0
			(0.1)		(0.3)								
*Alburnus alburnus*		0.005	0.03	0	0	0	0	0	0	0	0.4	0	0
			(0.5)								(1.6)		

NNS occurred in 33% of the sites, representing nearly 11% of both the total mean density (3.58 ind./100 m^2^, SD = 12.54) and number of species (0.54, SD = 0.94) per site. Although the absolute values may seem low, they represent an important percentage of the total density and number of species, as Mediterranean streams usually show low species richness and density per site. Biomass values were not shown or included in the analysis, as they followed the density pattern and were therefore redundant.

There were significant differences in the density (F _(11,380)_ = 8.04; *P*<0.001) and proportion of non-native species (F _(11,380)_ = 9.20; *P*<0.001) among river types considering the total human pressure as a covariable in the analysis. NNS did not occur in the mountain river type or in the southern chalk river type. The small northern stream types, N1<100 km^2^, N3 and N4, registered very low densities, frequency of occurrence, and percentage of NNS (Table 4). N2 and S2 also showed low densities of NNS, but these species represented more than 15% of the fish assemblages in each site and registered high values of frequency of occurrence (f. oc.). N1>100 km^2^ and S1<100 km^2^ presented high values of occurrence and density of NNS, also representing more than 10% of total density and species richness. Littoral and southern river types S1>100 km^2^ and S3 showed the highest occurrence and abundance of NNS; in Littoral type, NNS represented almost 12% of the total density and 15% of the total species richness, S1>100 km^2^ and S3 registered percentages between 20% and 30%.

The most frequent and abundant NNS were *L. gibbosus* (mean density = 0.89 ind./100 m^2^, SD = 3.3; f. oc. = 0.21), *G. lozanoi* (mean density = 1.79 ind./100 m^2^, SD = 10.9; f. oc. = 0.14), and *G. holbrooki* (mean density = 0.54 ind./100 m^2^, SD = 3.1; f. oc. = 0.11) (Table 4). The remaining species registered low occurrences and abundances. *L. gibbosus* showed a wide distribution in both northern and southern river types - N1>100 km^2^, N2, L, and mostly southern river types (Table 4), which are associated with high annual temperature and conductivity, large drainage area, and low altitude (Table 1). *G. holbrooki* were the most abundant species in the southern river types, showing a distribution almost limited to these river types, while *G. lozanoi* was the dominant species in north/central river types, exhibiting high occurrence in river types with large drainage area and low altitude, especially in the north/centre region - N1>100 km^2^, N2, and L (Table 4). *C. carpio* and *M. salmoides* occurred in several river types, showing higher values in the south. *H. facetum*, *A. melas*, and *A. alburnus*, only occurred in the southern river types S1<100 km^2^, S1>100 km^2^, and S3 and *S. lucioperca* only occurred in N1>100 km^2^, all presenting very low frequencies of occurrence and abundance. *C. auratus* registered nearly vestigial occurrence in N1>100 km^2^, N2, and S3 (Table 4).

Overall, disturbed sites showed significantly higher occurrence (f. oc. = 0.46, *P*<0.001) and abundance (mean = 6.41, SD = 16.8, *U*_380_ = 12155.00, *Z* = -6.56, *P*<0.001) of NNS than undisturbed ones (f. oc. = 0.18; mean = 0.5, SD = 1.7). *M. salmoides*, *A. melas*, *S. lucioperca*, and *A. alburnus* only occurred in disturbed sites. All NNS but *H. facetum*, exhibited significantly higher occurrence (*P*<0.01) in disturbed sites. Fish density was significantly higher in disturbed sites only for the most abundant species, *L. gibbosus* (*U*_380_ = 15029.50, *Z* = 3.94, *P*<0.001), *G. lozanoi* (*U*_380_ = 15594.50, *Z* = 3.73, *P*<0.001), *G. holbrooki* (*U*_380_ = 14676.50, *Z* = 5.68, *P*<0.001), and *M. salmoides* (*U*_380_ = 17199.00, *Z* = 2.91, *P*<0.01). In all river types but N3, the occurrence of NNS was significantly higher in disturbed sites (*P*<0.01). The abundance and percentage of NNS were higher in human-disturbed sites in L, N1<100 km^2^, N1>100 km^2^, S1<100 km^2^, and S1>100 km^2^ (Table 4). The majority of river types that did not revealed significant differences between undisturbed and disturbed sites presented null or very low abundance of non-native fishes.

**Figure 2:**
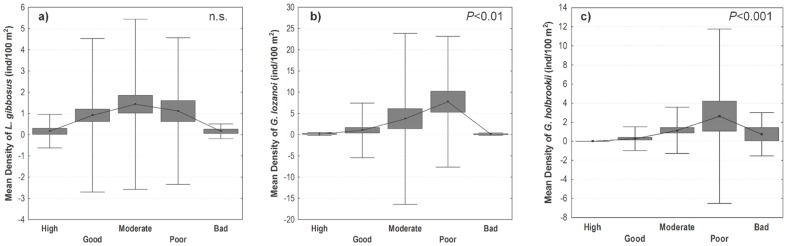
Response pattern of the most abundant NNS to human-induced pressure gradient, established according to 5 environmental quality classes: a) Mean density of *L. gibbosus*; b) Mean density of *G. lozanoi*; c) Mean density of *G. holbrooki*. (▪): Mean; box:+SE; whisker:+SD. Significance of ANCOVA results with total human pressure as a covariable is shown.

No significant correlations (Spearman's rank correlations IrI <0.5) were observed between the density of the most abundant NNS and total human pressure, as this relationship was not linear. A marked increase in fish density along the human disturbance gradient considering the environmental gradient (PCA1) as a covariable was observed, but there was a decrease in fish abundance in the extreme of the gradient, when the pressure was maximum (Fig. 2). This was particularly evident for *G. lozanoi* (F _(4,246)_ = 5.34, *P*<0.001) and *G. holbrooki* (F _(4,235)_ = 7.07, *P*<0.001), as mean fish densities reached the highest values in the poor quality class. The increase in fish density from moderate to poor quality classes was particularly marked for *G. holbrooki*. This species showed a comparatively higher mean density in the bad quality class and a smooth decrease between the poor and bad quality classes. *L. gibbosus* densities showed a considerable dispersion in each quality class. Nevertheless, the highest mean density was observed in moderate human pressure conditions, decreasing in the poor quality class and presenting very low values in the extreme of the pressure gradient (Fig. 2). Overall, the response of this species along the disturbance gradient was less marked (F _(4,343)_ = 0.30, *P*>0.05) than that observed for the other species.

### Relative importance of pure environmental and human pressure variables

**Figure 3:**
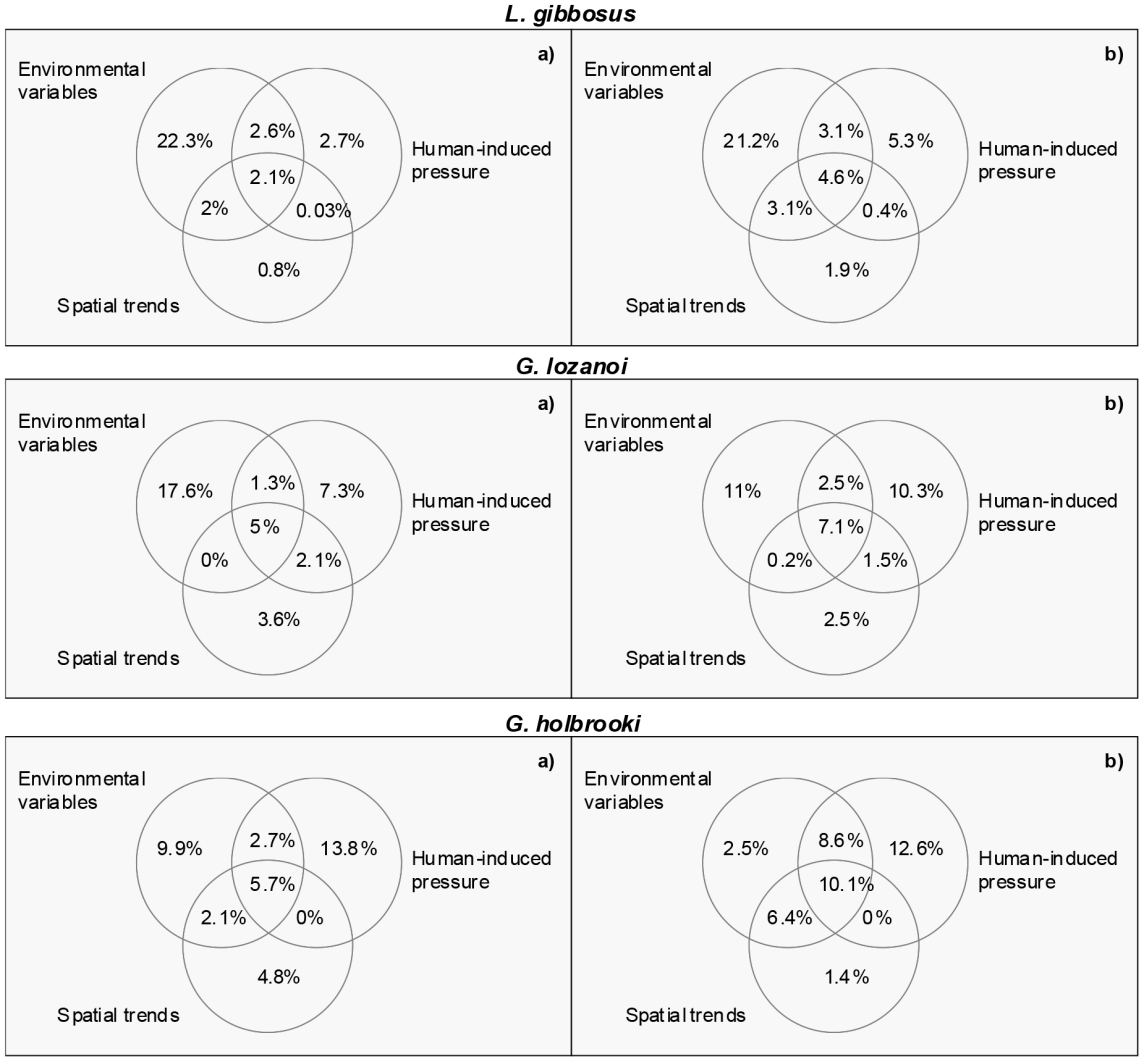
Veen diagrams showing the partition of the variance in the occurrence (a) and mean density (b) of the most abundant NNS explained by environmental variables, human-induced pressure and spatial trends.

The results obtained above showed that both environmental and human pressure variables play a determinant role in the occurrence and abundance of non-native species. Moreover, the performed analyses revealed the existence of significant covariation (*P*<0.001) between these two groups of variables. Therefore, the analysis of the relationship between the occurrence and abundance of the most abundant NNS and the environmental and human pressure gradients was complemented with a GLM approach in order to simultaneously evaluate the relative influence of environmental variables, human-induced pressure, and spatial trends. All GLMs showed over-dispersion parameters under the threshold limit of 1, and were therefore performed using binomial and Poisson distributions as previously described. For all the response variables, most of the explained variability was accounted for by unique, or “pure”, components. Shared effects of explanatory variables were responsible for a comparatively small proportion of data variability (Fig. 3). Together, environmental variables and human pressure accounted for the majority of the explained variation in the occurrence and abundance of the three considered NNS (31.7% to 40.2%) (Fig. 3).

The partition of the variance showed some differences in the relative influence of each variable set among the three NNS. The occurrence of NNS was mainly determined by environmental variables, whereas abundance showed a proportionally higher influence of human pressure variables. *L. gibbosus* occurrence and abundance revealed a high dependence on environmental variables. *G. lozanoi* presented a balanced influence of environmental and pressure variables. *G. holbrooki* showed a strong influence of human pressure on both occurrence and abundance (Fig. 3). The results suggest that these three species have different levels of tolerance to human pressure. In all models, spatial variables (autocorrelation) represented the smaller fraction of the explained variation in both occurrence (0.8% to 4.8%) and abundance of the three NNS (1.4% to 2.5%) (Fig. 3). A higher influence of these variables on the occurrence of *G. lozanoi* and *G. holbrooki* was observed, which is in accordance with a more restricted and concentrated distribution of these two species.

Overall, both regional and local explanatory variables were present in the best ecological models in environmental and human pressure GLMs (Table 5). Amongst environmental GLMs, drainage area, in particular, showed a significant reduction in deviance, and positively shaped all the response variables. Slope (positive coefficient) and percentage of riffles (negative coefficient) were ranked as the second most important predictors respectively in G. lozanoi and L. gibbosus occurrence and abundance. For G. holbrooki only mean annual temperature (positive coefficient) revealed a higher positive influence than drainage area. Concerning human pressure variables, toxicity and acidification levels were the most important predictors and were negatively related to the occurrence and abundance of the three NNS. For L. gibbosus, TSS and COD also assumed a significant positive influence. Although nutrient/organic loads were retained in both L. gibbosus human pressure GLMs, these variables did not present a relevant decrease of deviance. Total dissolved nitrogen (N) and urban area showed an important relation with G. lozanoi occurrence and density. Sediment load revealed a particular influence on G. holbrooki occurrence, presenting the highest deviance reduction in this model.

**Table 5:** Summary of the environmental and human pressure GLMs for *L. gibbosus*, *G. lozanoi* and *G. holbrooki* (response variables occurrence and density).

Species	Response variables	Models features	Models AIC	Explanatory variables	Type	Deviance reduction	Coef. sign	VIF	*P*
***L. gibbosus***	Occurrence	Binomial distribution		Drainage area	Env	76.31	+	2.14	***
		“logit” link function	274.7	% Riffles	Env	21.95	-	1.5	*
				TSS	Pres	21.93	+	1.09	*
			356.5	Nutrient/organic loads	Pres	3.06	+	2.09	*
	Density	Poisson distribution		Drainage area	Env	69.53	+	2.36	***
		“log” link function		% Riffles	Env	28.44	-	1.63	**
			430.9	Mean annual runoff	Env	7.66	-	1.78	*
				Toxic/acid levels	Pres	18.99	-	1.44	*
				COD	Pres	13.08	+	1.31	**
				TSS	Pres	5.66	+	1.12	*
			496.1	Nutrient/organic loads	Pres	2.68	+	2.19	*
***G. lozanoi***	Occurrence	Binomial distribution		Drainage area	Env	38.40	+	2.21	**
		“logit” link function	216.3	Slope	Env	11.22	+	1.61	*
				Dissolved N	Pres	17.62	+	1.42	**
				Toxic/acid levels	Pres	13.53	-	1.92	*
				COD	Pres	5.39	-	2.27	*
			236.1	TSS	Pres	4.31	+	1.29	**
	Density	Poisson distribution		Drainage area	Env	26.26	+	2.73	**
		“log” link function	286.4	Slope	Env	13.73	+	1.69	*
				COD	Pres	15.06	-	2.17	**
				Urban area	Pres	11.41	+	2.62	*
				TSS	Pres	4.94	+	1.22	*
				Toxic/acid levels	Pres	6.16	-	1.69	*
			293.38	Land use	Pres	6.13	-	2.43	*
***G. holbrooki***	Occurrence	Binomial distribution		Mean annual temperature	Env	22.88	+	2.12	*
		“logit” link function		Drainage area	Env	14.87	+	2.38	**
			186.1	% Riffles	Env	7.86	-	1.44	*
				Sediment load	Pres	22.06	+	2.31	***
			186.7	Toxic/acid levels	Pres	13.21	-	1.98	***
	Density	Poisson distribution		Mean annual temperature	Env	26.06	+	2.25	*
		“log” link function	247.3	Drainage area	Env	9.48	+	2.29	*
				Toxic/acid levels	Pres	42.29	-	1.77	**
				Sediment load	Pres	9.70	+	1.36	*
			240.8	Artif. lentic water bodies	Pres	6.40	+	2.75	*

Explanatory variables type: environmental (Env) and human-induced pressure (Pres). Significance levels (*P*) at

*P<0.05.

**P<0.01.

***P<0.001.

For all the models produced, values of VIF were very low (Table 5), indicating the lack of significant multicollinearity between predictors, ensuring that no covariation exists among the variables included in the models, and therefore ensuring the reliability of the conclusions arising from the results.

## Discussion

Non-native fish present in Portuguese rivers have different histories. Although rapid spread is possible in a short period, the timing and location of NNS introductions is relevant to the expansion of species distribution and abundance, which explains the relatively narrow distribution and abundance of *A. melas*, *A. alburnus*, and *S. lucioperca*, all recently introduced [9]–[10], [51] at the date of this study, compared with other NNS introduced earlier. The most frequent and abundant species were *L. gibbosus*, *G. holbrooki and G. lozanoi*. In southern rivers the most common and abundant NNS were the former ones while *G. lozanoi* dominated the northern rivers. As such, the discussion will mainly focus on these NNS, which presented the higher invasive potential and are totally established in Portuguese continental waters for more than forty years.

A striking fact regarding the distribution of NNS in this study was their absence from mountain and southern chalk river types. Mountain rivers and small highland streams, with high slope and energy and low productivity (such as M, N4, S4 and N3), seem to be much less vulnerable to invasion by the set of introduced species. The upstream reaches of most Mediterranean basins experience strong seasonal patterns in their environmental conditions. In winter and spring, these streams present high flows whereas in summer they are frequently reduced to small isolated pools. This hydrological regime may prevent the invasion of NNS which are poorly adapted to high discharge events, including flash flows (e.g. [52]–[53]). Conversely, NNS occurrence and abundance were particularly meaningful in river types with medium and large drainage areas, both in the northern “cool–warm water” and in the south. The high proportion of non-native fishes in the middle and lower reaches of Mediterranean-climate rivers was also reported in other studies (e.g. [54]–[56]). In general, the pattern of NNS distribution and abundance among river types followed the pattern of human-induced disturbance. Although human-induced disturbance occurred among all river types, some areas exhibited higher degradation. Overall, the sites located in littoral regions, both in the centre-north and in the southern region, which are associated with higher human density, presented higher degraded conditions and higher occurrence and abundance of non-native fishes. The least disturbed river types were located in higher altitude regions and with small drainage areas, where NNS were absent or present in very low densities. Most of those streams are located in isolated areas with difficult human access, far from the main human pathways. Remote areas with low human disturbance receive much lower propagule pressure than areas hosting immense human settlement [57].

Sites with higher human pressure exhibited significantly higher occurrence, abundance, and percentage of NNS. This pattern was observed in all river types and for all species, excepting those with very low abundance of NNS and *H. facetum*. Several species (*A. alburnus, A. melas, S. lucioperca*) occurred exclusively in impaired sites. The response of the most abundant NNS to the human pressure gradient was shaped by a marked increase in their abundance along the quality classes. Though the same general pattern was observed for the three species, *G. lozanoi* and *G. holbrooki* were particularly responsive to degradation, reaching the highest abundances in the poor quality class.

Non-native freshwater fish have commonly been documented to succeed in degraded aquatic habitats in many areas of the world [28], [58]–[61]. Disturbed systems and communities may attract biological invasions more than pristine ecosystems due to the redistribution of space and energy resources and may promote new vacant niches for the most adaptable and tolerant invaders [11], [62]. One of the most important human pressures in Mediterranean-climate rivers is related to nutrient and organic loads, which under the highly favourable climatic conditions increase the aquatic productivity and food availability. The resource availability theory argues that invasibility is directly influenced by nutrient enrichment and resource availability [11], [63], which may facilitate biological contamination by reducing resource limitation and therefore competition. The high proportion of NNS in medium and large lowland rivers where productivity is naturally higher corroborates this hypothesis. Moreover, these river types (Littoral, N1>100 km^2^ and S1>100 km^2^) are also the most affected by hydrological disturbances, flow regulation, connectivity loss, and dam construction. Dams typically create lentic conditions that favour non-native fishes, thus presenting higher abundance in regulated streams than in unregulated ones [64]–[65]. These river types are particularly vulnerable to non-native fish invasions, as some species, namely *L. gibbosus* and *G. holbrooki*, prefer enriched lentic habitats (e.g. [53], [66]).

This study reveals that natural landscape/environmental drivers and human-induced disturbance are both important factors determining the distribution and invasiveness of NNS, and their relative importance vary from species to species.

The occurrence of *G. lozanoi* was mostly explained by environmental variables, while its abundance was equally determined by landscape/habitat and human-disturbance factors. In the Portuguese continental waters *G. lozanoi* populations were particularly abundant in N1>100 km^2^ and littoral river types which is consistent with previous work associating this species to lowland rivers with slow flow [67]. Being absent from high altitude rivers and warm waters, this species occurrence was positively related to slope and drainage area (larger areas providing water flow all year long). For this reason, *G. lozanoi* does not occur in the warmer southern and central rivers, which present low or no flow during summer. The species abundance was positively correlated with human-induced hydrological disturbance, habitat modification, water quality degradation (TSS, COD, acidification) and agricultural areas. Other studies also evidenced this species response to anthropogenic hydrological disturbance [64], [68].

*G. holbrooki* is mainly present in southern lowland river types (S1>100 km^2^, S1<100 km^2^ and S3) with the highest water temperature and conductivity. This species occurrence was positively correlated with temperature and drainage area. Although G. *holbrooki* is able to withstand wide temperature ranges, it prefers warm water temperatures [69]–[70]. Conversely, the lower temperatures of the northern rivers could limit the species proliferation, due to the effect of latitude on life-history traits, namely the reproductive potential [71]. At the habitat scale, *G. holbrooki* was negatively associated with riffles, that is, it displays a preference for standing or slow flowing waters rich in organic detritus and muddy sediments. High river discharges tend to displace individuals and eliminate populations [52], [58], [72], as this species has no swimming abilities and behavioural response to fast flowing waters [73]. The invasiveness of *G. holbrooki* seems to be mostly determined by human-induced disturbance factors. Its abundance was strongly related to indicators of disturbance describing local in-stream habitat modifications and organic and sediment loads. The response to degradation is partially related to the species tolerance to a wide range of environmental conditions, including pH (from 3.9 to 8.8, [74]) and dissolved oxygen (0.28 mg/L, [69]–[70]. The ability to tolerate low dissolved oxygen enables it to survive in anoxic eutrophic waters with high organic and nutrient loads, and it is often the only fish species present in these water bodies (Ilhéu pers. obs.). Moreover, this species is also tolerant to a wide range of pollutants, including organic wastes, phenols, pesticides, and heavy metals, due to the species' phenotypic plasticity [75]–[76].

*L. gibbosus* is one of the most widespread NNS in Portugal and a very successful invader. This species was particularly frequent and abundant in warm water rivers of southern and central Portugal – littoral and southern river types, with the exception of southern mountain rivers – while in the north it occurred mainly in larger rivers (N1>100 km^2^ and N2). *L. gibbosus* responded positively to a certain degree of anthropogenic disturbance, namely hydrological and habitat modification, and nutrient and organic loads, probably because these conditions lead to an increase in suitable habitats and also offer feeding advantages [77]. The intermediate tolerance of *L. gibbosus* to degradation, also reported by other authors [78], supports the idea that human-induced disturbance is not a requisite for successful invasion by all introduced species [79]–[80], and that a particular site may contain introduced organisms simply because they were introduced there and natural environmental conditions were favourable to their establishment. Indeed, this species abundance and distribution was mostly explained by pure environmental variables; its density was positively related to drainage area and negatively related with runoff and percentage of riffles, which is consistent with its higher occurrences and abundance in the warm water lowland rivers. Sunfishes exhibit low ability to withstand high current velocities and are often reduced in number by flood events [73], [81]. Differences in swimming ability or behavioural response to high current velocities between native and non-native species may be the mechanism responsible for the observed differential removals [65]–[66]. Furthermore, *L. gibbosus* is known to be invasive in some southern and central European locations but not in England or Norway [82]–[84], being the climatic conditions the limiting factor in the species invasiveness [85]. This illustrates the high vulnerability of the warm water rivers of southern Europe, where environmental conditions tend to favour the proliferation of this type of species, being high flows the major constraint.

The invasive potential of NNS seems to be determined by different environmental factors that may interact direct and indirectly. However, other factors may also explain the success of many NNS invasions, namely the spatial structure and the biota features, including species life-histories and interspecific interactions [86]–[88]. Thus further studies integrating all these factors should be performed in order to enhance the ability to predict the vulnerability of ecosystems to invasive species.

## Conclusions

This study reveals that natural landscape/environmental drivers are major factors determining the distribution of non-indigenous species in Portuguese rivers, while their abundance seems to be largely promoted by human-induced disturbance. The importance and role of different invasion factors are context dependent, because of the interaction between species traits and environment, regarding habitat, climate match, and human disturbance, and thus the river type approach seems to be useful to predict NNS invasiveness. This study also stresses the high vulnerability of the warm water lowland river types to invasion by NNS where conditions are amplified by human-induced degradation. Although some warm water species may be present in most basins (e.g. *L. gibbosus*), they become successful invaders mainly in large lowland rivers, particularly in the south. In contrast, high energy and low productivity rivers, typically at higher altitudes, seem to resist better to the invasion of NNS even when subjected to anthropogenic disturbance. These are relevant issues for assessment of the ecological status of aquatic systems, river basin management and river rehabilitation programmes.
